# Dendrimer-Capped Nanoparticles Prepared by Picosecond Laser Ablation in Liquid Environment

**DOI:** 10.3390/molecules14093731

**Published:** 2009-09-22

**Authors:** Emilia Giorgetti, Anna Giusti, Francesco Giammanco, Paolo Marsili, Simona Laza

**Affiliations:** 1 INSTM and Istituto dei Sistemi Complessi, Consiglio Nazionale delle Ricerche, Via Madonna del Piano 10, 50019 Sesto Fiorentino, Firenze, Italy; 2 Department of Physics "E. Fermi", University of Pisa, Largo Bruno Pontecorvo 3, 56127 Pisa, Italy

**Keywords:** dendrimers, laser ablation, metal nanoparticles, CdSe, photofragmentation

## Abstract

Fifth generation ethylendiamine-core poly(amidoamine) (PAMAM G5) is presented as an efficient capping agent for the preparation of metal and semiconductor nanoparticles by ps laser ablation in water. In particular, we describe results obtained with the fundamental, second and third harmonic of a ps Nd:YAG laser and the influence of laser wavelength and pulse energy on gold particle production and subsequent photofragmentation. In this framework, the role of the dendrimer and, in particular, its interactions with gold clusters and cations are accounted.

## 1. Introduction 

Dendrimers are monodisperse polymers characterized by a well defined structure that resembles a tree, a multifunctional surface and internal cavities [1]. Many potential applications of dendrimers are based on these properties. In fact, they are widely exploited either as model systems for polymers, or as materials with novel and tunable properties for biomedical and industrial purposes. The presence of internal cavities opens the way to the possibility of encapsulating, in the macromolecule interior, guest molecules as genetic materials or chemical markers [2,3,4,5,6], that can be subsequently released by a chemical reaction, like hydrolysis, or by photochemical modifications of the dendritic surface [7]. Dendrimers are also small enough to enter into the cells and their presence can enhance the therapeutic effect of some drugs [8,9]. For example, they can increase the solubility of drugs in the aqueous environment of biological tissues [10,11], or can be used as coating agents to protect or deliver drugs to specific sites, or also as time-release vehicles for biologically active agents. Besides biomedical applications, dendrimers can be used to improve many industrial processes, while the combination of high surface area and high solubility makes them useful as nanoscale catalysts [12,13]. Thanks to their multifunctional surface, dendrimers can be self assembled onto differently functionalized surfaces to obtain regular arrays and to control the spacing of interior guest molecules [14]. This can allow a fine tuning of magnetic, optical and electronic properties of the composite. 

The most widely known class of dendrimers are the PolyAMidoAMines (PAMAM). They are characterized by three distinct features: an ammonia or ethylendiamine core, several multifunctional repeating (*N*-(2-aminoethyl)propionamide) units attached regularly around the core and several end groups that are generally amino groups for a full generation dendrimer, or carboxylate groups for the half-generations. Among the different applications of PAMAM, an interesting one is its use as stabilizer for noble metal nanoparticles. Indeed, nanoparticles of noble metals are of great interest because of their potentialities into several rapidly developing fields, including tailor-made nanostructured materials for medicine and biosciences, drug delivery, *in vivo* cellular Raman spectroscopy and, thanks to their characteristic plasmon resonances, for photonics and information technology [15]. In general, in the case of gold or silver, the nanoparticles (Au or Ag NPs) can be produced in the form of colloidal suspensions in solution either chemically, or physically by pulsed laser ablation of a metallic target, the second procedure being advantageous in terms of purity and biocompatibility of the final products [16,17,18]. PAMAM has been widely exploited in the preparation of stable suspensions of metal (Cu, Au, Ag, Pt, Pd) nanoparticles by chemical reduction. In particular, the role of PAMAM generation and surface end groups on the architecture of the nanostructures, as well as of the concentration of precursor ions on nanoparticles size has been assessed by several authors. It has been shown that amino terminated PAMAM of lower generation (G2-G4) surrounds the metal nanoparticles thus acting as a stabilizer, while PAMAM of higher generation (G6-G10) acts as nanotemplate in forming host-guest structures. Generation 5, in contrast, exhibits an intermediate behavior [15].

On the contrary, at least to our knowledge, PAMAM had never been used for the synthesis of metal or semiconductors nanoparticles by laser ablation in liquid environments before our recent experimental reports [16,17,18]. Indeed, we found that its chemical structure is particularly suited for efficient trapping and stabilization of the different products extracted from the targets during the ablation process, i.e., atoms, small clusters and ions. This can permit to isolate the different species produced by the interaction of the laser pulse with the solid target, thus achieving a deeper insight into the different mechanisms involved in the laser ablation process. 

In this paper, we investigate the role of PAMAM dendrimers of generation 5 on the stabilization of metal (Au and Ag) or semiconductor (CdSe) nanoparticles (NPs) obtained by ps laser ablation of a target in water solutions. In particular, it is dedicated to extensive report of the results, which we have recently obtained on the preparation and characterization of PAMAM G5-capped AuNPs: i) We discuss the role of laser wavelength and energy per pulse in the ablation of the gold target; ii) we show that it is possible to control the size of the NPs by irradiating pre-formed NPs suspensions with green or UV laser pulses, thanks to the onset of photofragmentation and iii) we study the fluorescence of different NPs suspensions in order to evidence the contribution of Au quantum dots to their emission. We also show how the methods developed for preparation of PAMAM G5-capped AuNPs and the potentialities of the dendrimer as a particle stabilizer can be transferred to other systems. For this purpose, we illustrate preliminary experimental results, which we have very recently obtained in the case of laser ablation of Ag or CdSe targets in PAMAM G5/water solutions.

## 2. Results and Discussion 

### 2.1. PAMAM G5-capped AuNPs prepared with different ablating wavelengths

#### 2.1.1. Preparation

The mechanism of laser ablation of a metallic target and its dependence on laser wavelength and pulse duration is not clear yet. In general, it is determined by different competing effects, such as multiphoton absorption, thermoionic emission and melting, whose relative importance is difficult to establish. However, once the material is extracted from the target, particle formation and growth in liquid environment can be explained in terms of the dynamic mechanism first proposed by Mafunè and co-workers [19]. A plasma plume is formed by laser pulses in close vicinity to the target. The plume contains metal atoms, clusters and cations, which tend to aggregate rapidly into small seeds. The seeds continue to increase slowly by assembling other atoms and clusters provided by diffusion mechanisms, until the growth is stopped either by depletion of ablated species or, if stabilizing molecules are present in the suspension, when the surface of the nanoparticle is coated by the stabilizer. Hence, in general, particle dimensions can be tuned by both controlling the density of ablated species (which can be done by adjusting the laser fluence) or the concentration of the stabilizing agent. 

**Figure 1:**
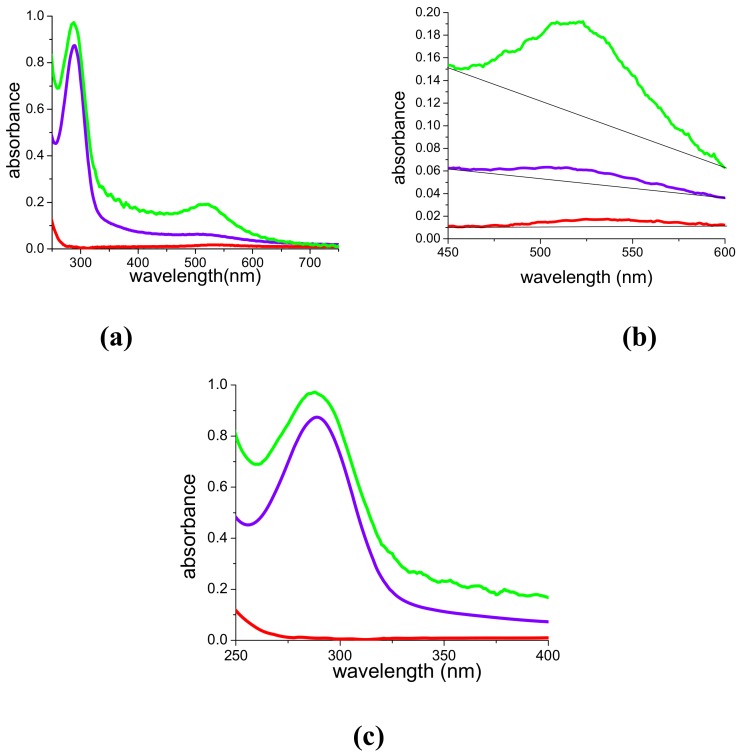
**(a)** UV-Vis spectra of PAMAM G5-capped suspensions of AuNPs produced for 16 min, with 5 mJ pulses at 1064 nm (red curve), 532 nm (green curve) and 355 nm (violet curve). Optical Path length OPL = 2mm. **(b)** and **(c)**: magnification in the plasmon and UV regions, respectively.

We prepared AuNPs by ablating a gold target in PAMAM G5 aqueous solutions with 1,064, 532 and 355 nm pulses. In all cases we obtained stable, wine-red samples containing AuNPs. Figure 1a shows complete UV-Vis absorption spectra corresponding to three samples obtained by 16 min ablation with 5 mJ pulses and different wavelengths. Figure 1b shows a magnification of the spectral region corresponding to the plasma resonance of the nanoparticles. The plasmon band is well resolved only for the sample obtained with 532 nm pulses (green curve), while in the two other cases it is very weak. Figure 1c, instead, shows a magnification of the spectra in the UV region. In this case, while the sample obtained with infrared (IR) light (red curve) does not exhibit any spectral feature, the two other samples exhibit an intense absorption band centered around 290 nm. 

The absorption spectrum of AuNPs-containing suspensions, in the case of sufficiently small (below 30-40 nm radius) and non-interacting spherical particles, is the result of two contributions: interband absorption of *d* electrons, which are promoted to *s-p* orbitals and collective oscillations of conduction electrons, i.e., dipolar plasmon resonances. The first contribution dominates at high energy, namely above 3 eV (i.e., ~350 nm), while the second one is responsible of the absorption band in the green region of the spectrum [20]. For this reason it is common practice to use the absorption of a sample at 400 nm as an indicator of gold concentration. 

According to Mie’s theory [21], the plasmon band associated to a single Au particle of radius R_NP_ appears only when R_NP_ is sufficiently large (radius R_NP_ > 0.5 nm). Its intensity is proportional to the particle volume, while the width broadens and tends to be swallowed by the interband contribution to the electronic spectrum in the case of particles approaching nm size. In the case of many identical and non interacting particles, the final absorption is obtained by multiplying the single particle contribution by the concentration.

Such considerations permit a first interpretation of the spectral differences observed in Figures 1a,b. In the case of green and IR ablation, although the total energy released on the target and the laser fluence are the same (5 mJ for 16 min and 0.3 J/cm^2^), the absorbance at 400 nm is negligible for the sample obtained with IR pulses and 16 times smaller than that obtained with 532 nm pulses, suggesting a very small Au concentration in the sample, namely a very inefficient ablation process. As a matter of fact, we have found that the energy of 5 mJ per pulse is very close to ablation threshold for 1,064 nm light in the ps time regime [16]. In contrast, if we compare the violet and green curves, which were obtained with the same energy but different laser fluences (0.3 J/cm^2^ at 532 nm and 0.15 J/cm^2^ at 355 nm), we observe that the most striking difference regards the visibility of the plasmon band. The poor visibility of the plasmon obtained with UV ablation suggests reduced dimensions of the nanoparticles, while the strong absorption at 400 nm suggests the presence of a high concentration of sub-nanoclusters. 

**Figure 2:**
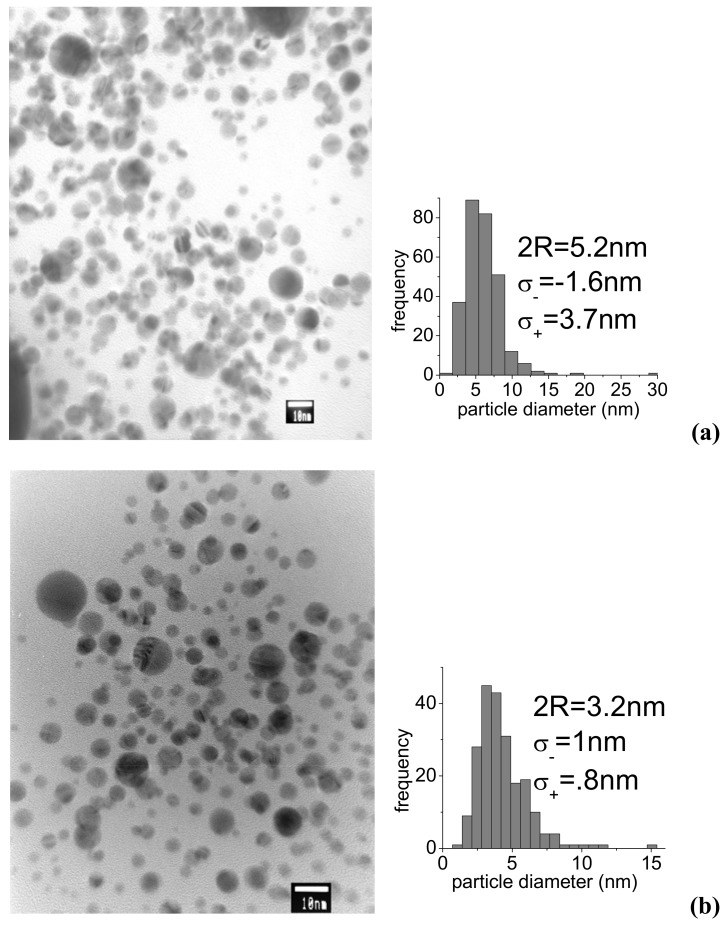

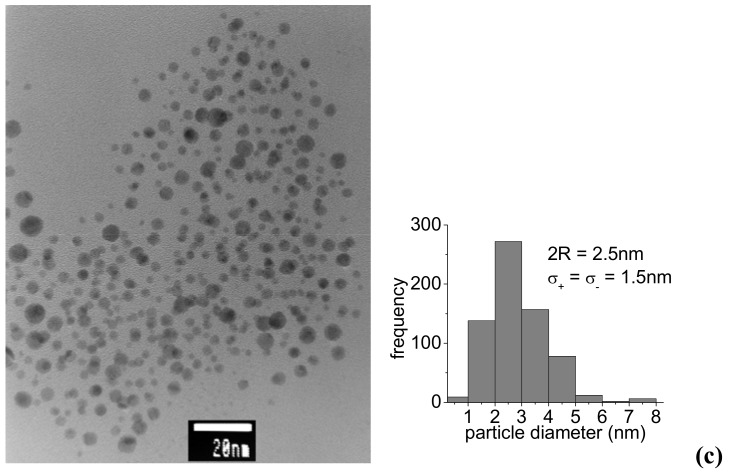
TEM images and statistical distribution of particle size for the samples of Figure 1: **(a)** 1,064 nm, 5m J, 16 min; **(b)** 532 nm, 5m J, 16 min; **(c)** 355 nm, 5m J, 16 min.

Such dimensional considerations are confirmed by the TEM analysis of the samples of Figure 1, which is shown in Figures 2a-c. The biggest dimensions and dispersivity were obtained with IR pulses (Figure 2a). In this case we found an average diameter 2R_NP_ = 5.2 nm and a distribution characterized by half widths σ_+_ = 3.7 nm and σ_-_ = 1.6 nm. In the 532 nm case (Figure 2b), we obtained 2R_NP_ = 3.2 nm and a statistical distribution with σ_+_ = 0.8 nm and σ_-_ = 1 nm. The lowest particle dimensions were found with UV ablation (Figure 2c), with 2R_NP_ = 2.5 nm and a statistical distribution characterized by σ_+_ = σ_-_ = 1.5 nm. 

#### 2.1.2. Control of dimensions

**Figure 3:**
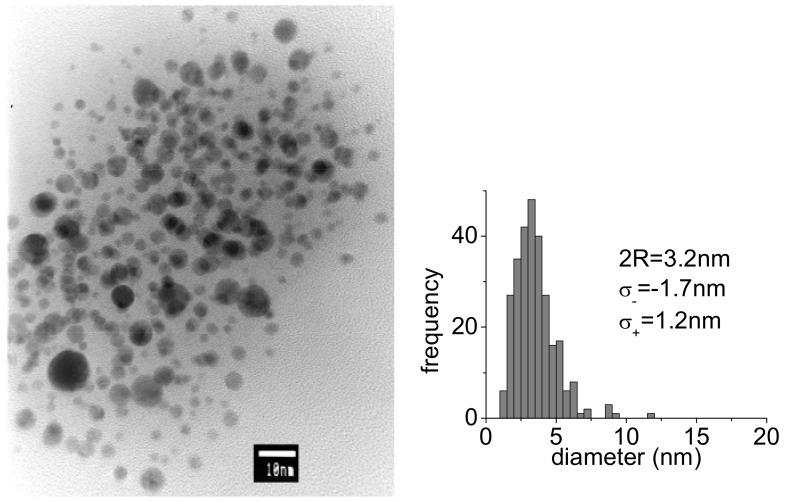
TEM image and statistical distribution of particle size for PAMAM G5-capped AuNPs produced with 1,064 nm, 15 mJ.

A way to control particle dimensions is the proper choice of the concentration of stabilizing molecules. In general, lack of stabilizing molecules leads to increased particle size. However, in our case, a reduction of PAMAM G5 concentration down to a factor of 20 led to a negligible variation, namely only a 10% increase of particle diameter. Bigger changes were observed at 1,064 nm by increasing the energy per pulse. Figure 3 shows the TEM picture of a typical sample of AuNPs obtained with 15 mJ 1064 nm pulses. A comparison with Figure 2a shows a roughly 40 % decrease of particle diameter, which reduces to 3.2 nm and a considerable narrowing of the statistical distribution with half widths of σ_+_ = 1.2 nm and σ_-_ = 1.7 nm. This is probably related to different ablation mechanisms and suggests that, at higher energy per pulse, and correspondingly higher fluence, material extraction by multiphoton absorption dominates over heating effects. 

A better control of particle size and dispersivity can be obtained by post irradiating PAMAM G5-stabilized-AuNPs with ps pulses at 532 and 355 nm. Indeed, at these wavelengths an efficient fragmentation process occurs, which permits both size reduction and narrowing of dispersivity of existing particles [17,18]. The presence of PAMAM G5 molecules permits efficient stabilization of the fragments and production of stable suspensions characterized by smaller nanoparticles and by a high concentration of sub-nm-sized particles and gold clusters or cations, which do not re-aggregate. Post-irradiation with either 532 or 355 nm wavelengths for sufficiently long time intervals permits complete bleaching of PAMAM G5-capped AuNPs suspensions with no evidence of precipitate. The photofragmentation effect also explains the smaller particle size typically observed in the samples prepared with 532 or 355 nm pulses, with respect to those prepared with 1,064 nm pulses. Indeed, when ablating with 532 or 355 nm light, the ablation and fragmentation processes coexist, while at 1,064 nm only ablation takes place, without any effect on the size of existing particles.

**Figure 4:**
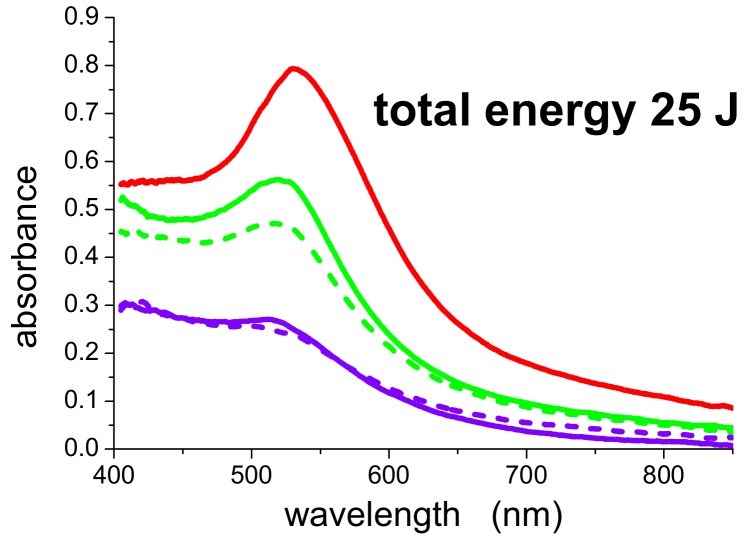
On-line UV-Vis spectra of a suspension of AuNPs obtained by ablation with 1,064 nm, 15 mJ before (solid red curve) and after post-irradiation with different wavelengths. Green curves: 532 nm, 5 mJ, 5,000 shots (solid) and 10 mJ, 2,500 shots (dashed). Violet curves: 355 nm 5 mJ, 5,000 shots (dashed) and 10 mJ, 2,500 shots (solid). OPL = 1 cm.

We studied the dependence of the photofragmentation process on wavelength and on pulse energy. At first glance, as already described in [17], the 532-nm-photofragmentation process is non linear and involves simultaneous absorption of two photons, while the 355-nm-photofragmentation is i) more efficient than the 532 nm one and ii) involves a single photon [18]. Such characteristics are illustrated in Figure 4, which shows a comparison between the photofragmentation process occurring at 532 and 355 nm. It refers to a suspension prepared with 1,064 nm, 15 mJ and post irradiated with 532 nm (5 mJ, 5,000 shots or 10 mJ, 2,500 shots) or 355 nm (5 mJ, 5,000 shots or 10 mJ, 2,500 shots). In all four cases the total energy released in the samples is the same, namely 25 J. However, the 532-nm-postirradiated suspensions (solid and dashed green curves) exhibit different plasmon bands, depending on the energy per pulse, the most energetic pulses being more efficient in the plasmon bleaching process. In contrast, the 355-nm-postirradiated suspensions (solid and dashed violet curves) exhibit identical spectra, with no substantial difference between the efficiency of 5 mJ or 10 mJ pulses. We evaluated that, in this case, the photofragmentation process is about 2.5 times more efficient with 355 nm pulses with respect to 532 nm pulses [18]. 

A thorough study aimed at providing a theoretical explanation for this behavior is currently in progress [22]. In general, under our experimental conditions, evaporation of the particles due to lattice heating above the melting point can be excluded and the photofragmentation process of PAMAM G5-capped AuNPs is due to the contribution of three different effects: i) two-photon ionization from the Fermi level; ii) thermoionic emission and iii) heating of conduction electrons above the Fermi level with subsequent one-photon extraction [22]. The first mechanism is found to be dominant in the case of 532 nm pulses, while the second and third one play a comparable role in the case of 355 nm pulses, leading to a net almost linear dependence. At both wavelengths, photoextraction of electrons causes accumulation of excess positive charges onto the particle and subsequent instability with progressive layer by layer peel-off or coulombian explosion [22].

To better stress the role of the dendrimer on the photofragmentation process, we repeated such experiments in different environments and found completely different results. For example, post-irradiation with 532 nm ps pulses of a suspension of AuNPs in pure acetone, which is a very efficient stabilizing solvent, does not permit the bleaching of the plasmon. Only a slight change of the plasmon band is observed, with some degree of bleaching, indicating some rearrangement of particle dimensions and statistical distribution. But, when the laser is switched off the particles re-aggregate and the initial plasmon shape and absorption are re-established. It means that, in this case, the products of fragmentation are not stable and tend to reproduce the initial NPs.

#### 2.1.3. Production of sub-nanoclusters and Au^3+^ by 532 and 355 nm irradiation

**Figure 5:**
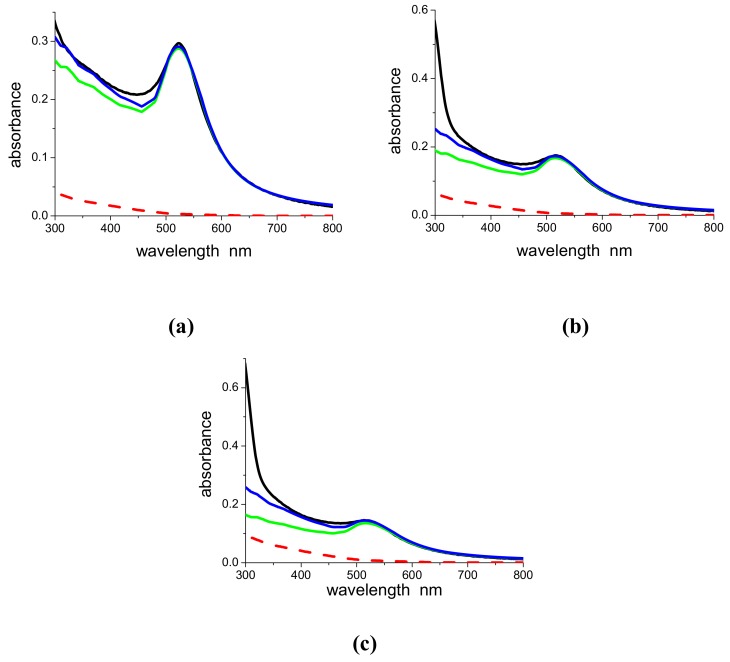
Simulations of UV-Vis spectra of PAMAM G5-capped AuNPs produced with 1,064 nm, 15 mJ **(a)** and post-irradiated with 532 nm, 5 mJ, 9,600 shots **(b),** or with 532 nm, 10 mJ, 4,800 shots **(c).** Black curves: experimental spectra; blue curves: complete fit; green curves: Mie’s fit without the contribution of sub-nm clusters; dashed red curves: contribution of sub-nm clusters. OPL = 1 cm.

When compared with ps pulses at 1,064 nm, ps pulses at 532 and 355 nm lead to formation of smaller particles and to efficient fragmentation of those already existing. Both processes are also accompanied by the production of sub-nanoclusters, which cannot be observed by TEM analysis, but which considerably contribute to the spectroscopic features of the suspensions. Indeed, such sub-nanoclusters are always present in our suspensions, due to incomplete growth of seeds. However, post irradiation with 532 or 355 nm pulses greatly increases their density. With the aim of better clarifying this point, Figures 5a-c show the experimental UV-Vis spectra (black curves) and the corresponding theoretical fits (blue curves) for a suspension of AuNPs obtained with 1,064 nm, 15 mJ ablation (Figure 5a) and post-irradiated with 532 nm in different conditions (Figures 5b,c). The fits were performed by using a home-made computer code based on Mie’s theory [21], whose details are given in the Appendix. 

Each theoretical spectrum was obtained in the hypothesis that the particles are coated by a 5.4-nm-thick PAMAM G5 shell and immersed in an environment having the dielectric constant of water, i.e. 1.77. The spectrum is the sum of two contributions: i) AuNPs having dimensions and statistical distribution obtained by TEM analysis (green curves) and ii) sub-nanoclusters (red curves). In all cases the first contribution alone is not sufficient to obtain a satisfactory fit of the spectroscopic features. 

In the case of Figure 5a, we obtained a dielectric constant of 2.5 for PAMAM G5, a density of nanoparticles N_NP_ = 1.1 × 10^13^ cm^-3^, and a total density of atoms belonging to sub-nanoclusters N_at_ = 1.9 × 10^16^ cm^-3^. Sixteen min post irradiation of the suspension of Figure 5a with 5 mJ ps pulses at 532 nm (Figure 5b) led to a reduction in particle dimensions [17] and to a consequent increase of both their density (N_NP_ = 3.2 × 10^13^ cm^-3^) and that of the atoms belonging to sub-nanoclusters N_at_ = 3 × 10^16^ cm^-3^. The dielectric constant of PAMAM G5 also changes to a lower value, namely 2.2, suggesting the onset of a photodegradation process of the molecule. Post irradiation with the same total energy (48 J) but with more energetic pulses (10 mJ) led to a more dramatic change in the spectral characteristics and in the density of AuNPs (N_NP_ = 8 × 10^13^ cm^-3^) and background atoms pertaining to sub-nanoclusters (N_at_ = 4 × 10^16^ cm^-3^), while the dielectric constant of PAMAM G5 turned out to be again 2.2 (Figure 5c). Analogous considerations can be repeated in the case of post- irradiation with 355 nm pulses.

**Figure 6:**
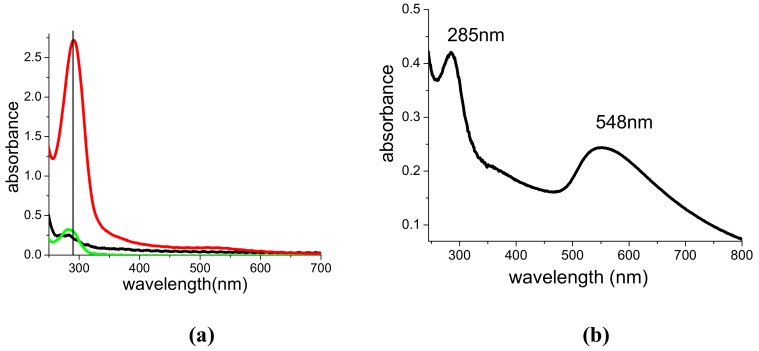
**(a)** UV-Vis spectra of PAMAM G5 in water multiplied by a factor of 10 (black curve), PAMAM G5 in water after irradiation with 532 nm, 15 mJ, 10,000 shots (green curve) and PAMAM G5-capped AuNPs obtained with 532 nm, 15 mJ, 10,000 shots (red curve). OPL = 1 cm **(b)** UV-Vis spectra of a suspension of PAMAM G5-capped AuNPs obtained by spontaneous reduction of a 1 mM solution di HAuCl_4_ in 3.8 mM PAMAM G5 in water. OPL = 1 mm.

Beyond the reduction of particle dimensions, and the production of sub-nm clusters, the preparation or the photofragmentation of PAMAM G5-capped AuNPs with 532 and 355 nm ps pulses causes strong modification of the electronic spectra, which cannot be explained with simple electromagnetic theory and which can be related to the release of gold cations and their interaction with the dendrimer. Indeed, Figures 1a,c and Figures 5b,c show the onset of an intense UV band, which does not appear when using infrared radiation, or different capping agents, such as SDS [17], or when performing the ablation in pure solvents. As reported in [18], this band, although very weak, is also generated by irradiation of pure PAMAM G5/water solutions by 532 or 355 nm ps pulses and can be assigned to photo degradation of the dendrimer [23]. In particular, as already observed in [17], it grows linearly with the total energy released in the solution. The intensity of the band increases considerably in presence of gold. As reported in Figure 6a for the case of 532 nm pulses, irradiation of the PAMAM G5 solution in the presence of the gold target, not only leads to formation of AuNPs, but also to a 10-fold intensification and a red-shift of the UV band, whose initial position was at 282 nm. A deconvolution of the spectrum shows that, in the presence of gold, the UV absorption is the result of the overlap of a dominant band peaked at 289 nm and a weaker one at 303 nm, which contributes around 10% to the total area. An analogous behavior is observed under UV irradiation. 

A band at 285 nm also appears under spontaneous reduction of HAuCl_4_ in PAMAM G5/water solutions, as reported in Figure 6b. In that case, the solution had been kept in the dark for several days, without addition of any specific reductant, such as NaBH_4_. Indeed, it is known that amino groups can reduce gold cations to AuNPs, as suggested in [24,25]. The reducing action of PAMAM G5 on HAuCl_4_ is demonstrated by the onset of the plasmon band, which is also accompanied by an UV band at 285 nm. Such behavior can be explained in terms of Au^3+^-catalized oxidation of the dendrimer [23,24,25,26]. In the light of these considerations, we can assign the UV absorption partly to a photo-oxidation of PAMAM G5 (the contribution around 285 nm), and partly to a Ligand to Metal Charge Transfer (LMCT) among PAMAM molecules and Au^3+^ cations originating in the fragmentation process of existing nanoparticles (the contribution around 300 nm). The gold cations complex with the PAMAM molecule and cannot reaggregate to form new nanoparticles, thus favoring the oxidation of the dendrimer and leading to an enhancement of the UV absorption. The photodegradation effect upon irradiation is further confirmed by the change of PAMAM dielectric constant, as obtained from the fit of UV-Vis spectra of Figure 5.

#### 2.1.4. Efficiency of the ablation process versus wavelength

**Figure 7:**
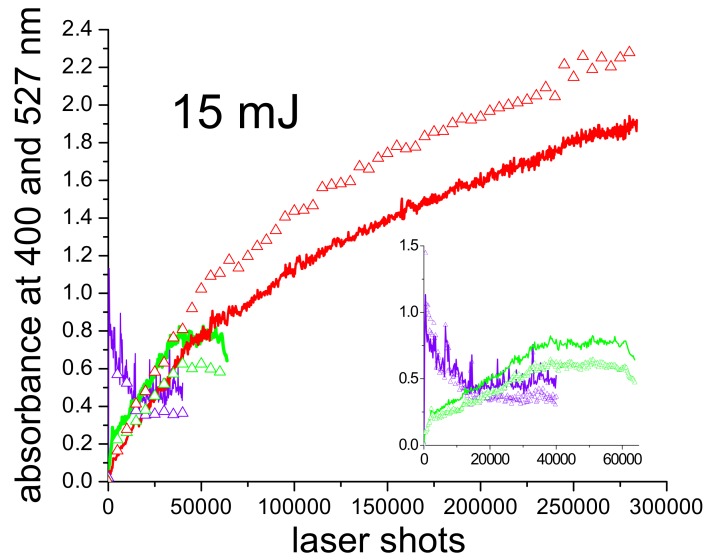
Absorbance at 400 nm (solid lines) and 527 nm (triangles) versus laser shots, during production of PAMAM G5-capped AuNPs with 15 mJ pulses at 1,064 nm (red), 532 nm (green) and 355 nm (violet). The inset shows a magnification of the zone corresponding to 0-60,000 laser shots. Fluence is 1 J/cm^2^ and OPL = 1 cm.

In the light of the previous considerations on the formation of sub-nm clusters or gold cations, which is observed particularly with 532 nm and 355 nm pulses, it is possible to explain another big difference among the three ablating wavelengths, which is already partially suggested by Figure 1, i.e., the rate of the process. For this purpose, we performed the ablation with all wavelengths at the same energy and fluence and monitored the absorbance (A) at 400 nm and in the plasmon maximum (527 nm) shot by shot. The results are reported in Figure 7, in the case of 15 mJ per pulse and 1 J/cm^2^ fluence, that are values well above threshold for all wavelengths, differently from the case reported in Figure 1. Solid lines and triangles in Figure 7 refer to absorbance at 400 nm and 527 nm, respectively. The data corresponding to 1,064 nm ablation are depicted in red in Figure 7. In this case, both material release in the suspension (A at 400 nm) and nanoparticle formation (A at 527 nm) exhibit a monotonic growth with laser shots, with a slow tendency to saturation around a value of A(527 nm) = 2.2. Absorbance in the plasmon peak is always larger than that at 400 nm, indicating that AuNP formation is very efficient and the amount of gold dispersed in the form of sub-nm clusters is negligible.

When ablation is performed with 532 nm pulses (green curve and triangles in Figure 7 and inset), the behaviour of A(400 nm) and A(527 nm) is no longer monotonic and we can distinguish four different phases: i) a fast growth during the first 2,000 shots (ablation of material from the target); ii) a slower growth up to about 38,000 shots (coexistence of efficient ablation from the target and photofragmentation); iii) a plateau up to 61,000 shots (balance between ablation and photofragmentation), which is followed by iv) a decay (prevalence of photofragmentation on ablation). Moreover, in this case, A(400 nm) is always larger than A(527 nm). It confirms that the production of sub-nm clusters or gold cations, either directly during the ablation of the target or as a second step during post-irradiation of existing particles, prevails over the process of nucleation and growth of the nanoparticles. In this case, the maximum value obtained for A(527 nm) is around 0.8, that is, a lower efficiency of the process for the production of AuNPs with respect to 1,064 nm ablation.

The data concerning ablation with 355 nm pulses are reported in violet in Figure 7 and inset. They are multiplied by a factor of 10 for better visibility. The maximum value for A(527 nm), in this case, is about 0.12, which is one order of magnitude lower than with the other two wavelengths. Such value is obtained after the first 500 shots and, afterwards, it rapidly decays due to the extremely efficient fragmentation process of the AuNPs, which are immediately destroyed by UV pulses after formation. The very low value of A(400 nm) suggests that both the gold extracted from the target and the products of the fragmentation are prevalently present in the form of cations, which do not contribute to A(400 nm). 

#### 2.1.5. Au^3+^-promoted fluorescence enhancement of PAMAM G5 molecules

As already noticed, 355 and 532 nm photofragmentation permits thorough bleaching of the suspensions, which turn perfectly transparent and clear, without evidence of any precipitate. This is an indication that the metal is still present, but in the form of sub-nm particulate, that is of gold clusters consisting of less than ~37 atoms. Such atomic aggregates are known as quantum dots (QD) and represent an intermediate state of the matter, between the atomic and the bulk one. Among the properties of QDs, fluorescence seems a particularly interesting one. Indeed, there are reports in the literature describing the fluorescence properties of gold QDs and claiming that such systems exhibit a high quantum yield and good resistance to bleaching [27,28]. In particular, PAMAM-capped AuQDs obtained by chemical reduction and formed by 5-13 atoms would be strongly fluorescent in the blue-green region of the spectrum [29]. Such observations have been questioned by other authors, who claim that the observed fluorescence comes from the dendrimer and not from the metal [30,31]. Therefore, we decided to measure the fluorescence of our bleached suspensions, in order to check the emission properties of the AuQDs contained therein and get a deeper insight on their possible interaction with the dendrimer. For this purpose, we bleached PAMAM G5-capped AuNPs suspensions with 532 nm pulses and different energies and compared their fluorescence spectra with those of PAMAM G5 aqueous solutions before and after irradiation in identical conditions. 

**Figure 8:**
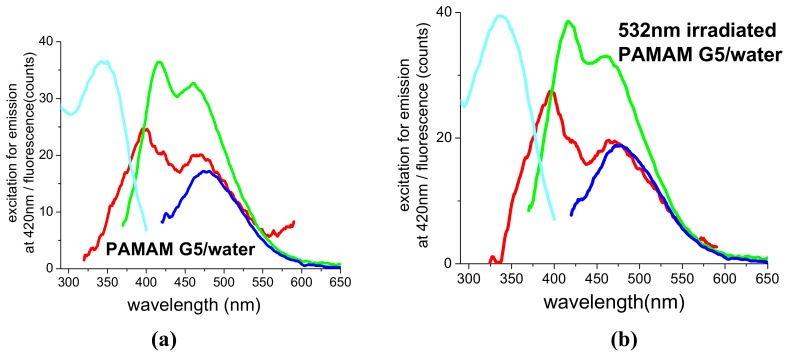

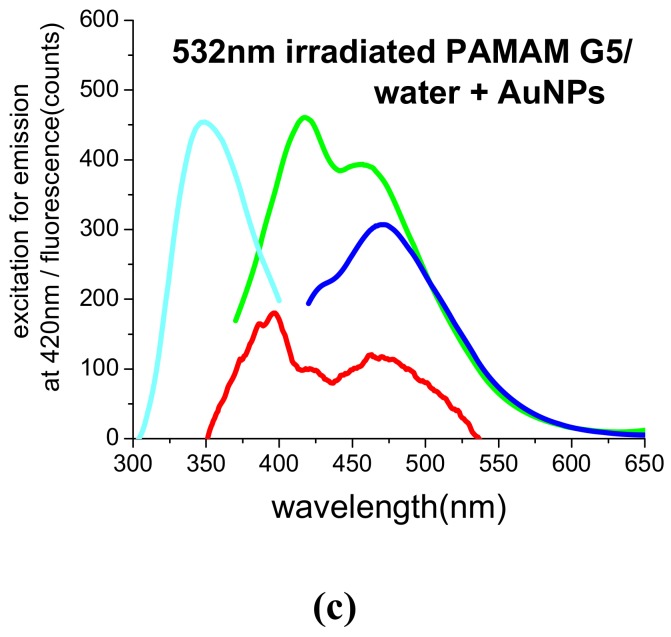
Excitation for emission at 420 nm (cyan curves) and fluorescence spectra of**:** PAMAM G5/water before **(a)** and after **(b)** irradiation with 532 nm, 8 mJ and 40,000 shots and **(c)** PAMAM G5-capped AuNPs after thorough bleaching with 532 nm, 8 mJ and 40,000 shots**.** Excitation wavelengths: 300 nm (red curves), 350 nm (green curves) and 400 nm (blue curves). Data corresponding to 300 nm excitation in (c) were multiplied by a factor of 10.

Figure 8a shows the fluorescence spectra of a PAMAM G5 solution under excitation at 300, 350, 400 nm. Several weak emission bands can be distinguished, while the excitation spectrum, also reported in the figure, is characterized by a single peak centred around 340 nm. As far as spectral features and intensity are concerned, such behaviour is not significantly modified by 532 nm irradiation, as reported in Figure 8b for the case of 8 mJ 532 nm pulses and 40,000 shots. The spectra of Figure 8 were deconvoluted. The results of such deconvolution, namely the position of the emission bands and their percentage contribution to the total fluorescence of each sample, are reported in Table 1. Deconvolution of the spectra of Figures 8a,b shows that, for excitation with 300, 350 and 400 nm the emissions are dominated by bands at 466, 470 and 480 nm, respectively. Figure 8c reports fluorescence and excitation spectra of a suspension of PAMAM G5-capped AuNPs after thorough bleaching with 532 nm pulses, 8 mJ and 40,000 shots. The initial suspension had been obtained with 1,064 nm pulses, 15 mJ and was characterized by 1.1 absorbance in the plasmon peak. From the data of Figure 8c and after proper deconvolution of the spectra (again reported in Table 1) and comparison with Figure 8b, we can infer that, due to the presence of AuQDs or Au^3+^ ions produced during the fragmentation.

- There is a more than 10-fold enhancement of the overall fluorescence for excitation at 350 or 400 nm and a 2-fold reduction of the overall fluorescence for excitation with 300 nm;
- There is a redistribution of the relative weight of the bands, depending on the excitation wavelength, but no evidence of new bands, which could be attributed to AuQDs;
- The most efficient excitation is observed with 350 nm. In particular, at this wavelength, the emission band at 470 nm is quenched, while the emission concentrates into a band at 454 nm, whose intensity was negligible in the case of Au-free PAMAM G5 solutions;
- For excitation at 400 mn the emission concentrates into the band at 470 nm, which is intensified by a factor of 300 with respect to the case of Au-free PAMAM G5 solutions.

According to what is reported in [32], the fluorescence of PAMAM dendrimers of different types is strongly dependent on the properties of the environment, such as pH, and can increase by orders of magnitude due to ageing or oxidation. Therefore, the fluorescence enhancement observed in our experiments can reasonably be attributed to the formation of Au^3+^ cations during the fragmentation of the AuNPs. Indeed, as we already noticed previously, Au^3+^ cations are trapped and stabilized by the dendrimer, acting as catalizers and favouring the oxidation of the molecule, as evidenced by the growth of the UV absorption around 290 nm. 

In the light of such considerations, we can exclude a direct contribution of AuQDs to the fluorescence signal, which is to be assigned thoroughly to the dendrimer. As a matter of facts, it is still unclear which part of the molecule acts as colour centre, i.e., the terminal groups or the inner cavities. According to ref. 32, PAMAM fluorescence must be attributed mainly to the interior of the molecule even if, on the basis of fluorescence life time spectroscopy, the authors do not exclude the existence of either more than one fluorescent moiety, or a single emitter into two distinct structural microenvironments. Such hypothesis could reasonably explain the rearrangement of the relative intensity of PAMAM G5 emission bands and the different enhancement factors, which we observed in the presence of gold. 

### 2.2. PAMAM G5 as an efficient stabilizer for AgNPs and CdSe quantum dots obtained by ps laser ablation

#### 2.2.1. PAMAM G5-capped AgNPs

**Figure 9:**
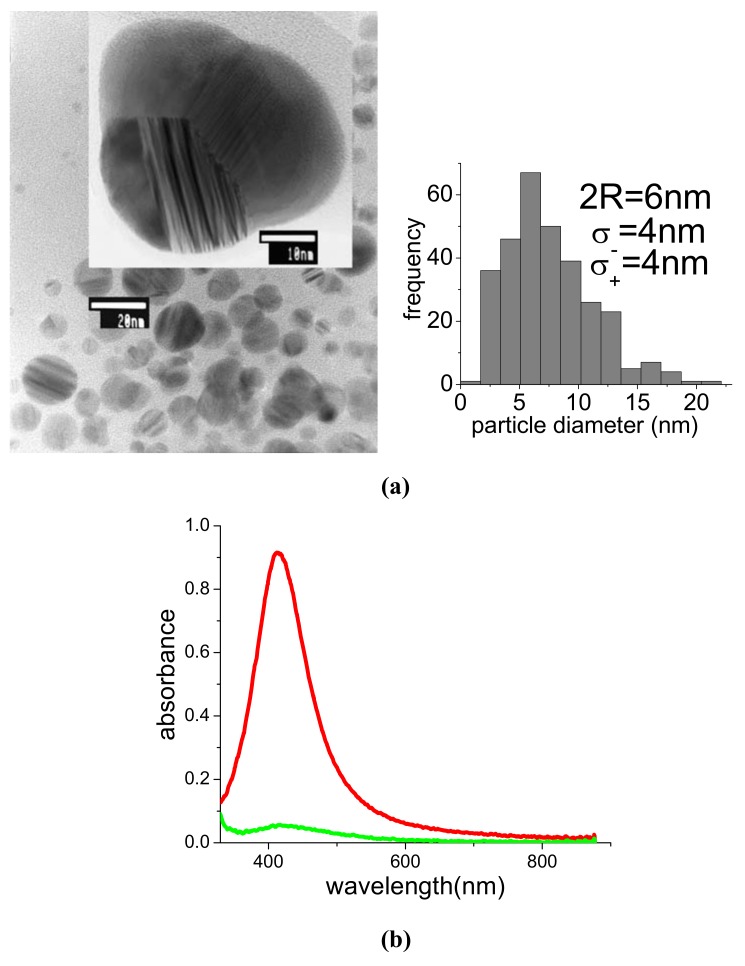
**(a)** TEM image and statistical distribution of particle size for a suspension of PAMAM G5-capped AgNPs produced with 1,064 nm and 15 mJ pulses. **(b)** UV-Vis spectra of the same suspension before (red curve) and after (green curve) post-irradiation with 532 nm, 8 mJ, 30,000 shots.

The same procedure adopted for preparation of PAMAM G5-capped AuNPs can be used in the case of silver. Figure 9a shows a TEM picture with corresponding statistical distribution obtained from a sample of AgNPs produced by ablation with 1064 nm and 15 mJ ps pulses. The average particle size was 2R_NP_ = 6 nm, with statistical distribution σ_+_ = σ_-_ = 4 nm. As in the case of gold, particles are mostly spherical. However, some of them exhibit irregular shapes, as the one shown in the inset of Figure 9a, which were not observed for AuNPs. Such irregularities are consistent with the pronounced tendency of Ag to coalesce in the form of polycrystalline nanostructures [33]. Figure 9b shows the absorption spectrum of the same sample of Figure 9a before (red curve) and after (green curve) 30,000 shots irradiation with 8 mJ pulses at 532 nm. The plasmon peak of the initial suspension was at 414 nm and could be efficiently bleached by photoirradiation, generating a transparent and clear suspension with no evidence of precipitate. This behaviour suggests that, as in the case of gold, post-irradiation permits control of particle dimensions and the dendrimer acts as a good stabilizer for Ag sub-nm clusters or cations, as well. 

#### 2.2.2. PAMAM G5-capped CdSe quantum dots 

There are few reports in the literature on the preparation of CdSe QDs by laser ablation. Indeed such systems, which are of great interest for several different applications, ranging from biology, to nanomedicine to photovoltaics [34], could take big advantage from the application of this technique as a fast and straightforward preparation method. Ref. [35] reports on ablation of a CdSe target with 532 nm ns pulses and in different solvents (water, acetone, ethanol and acetonitrile). The authors succeeded in the preparation of the QDs, but they did not observe any fluorescence. In contrast, ref. [36], which describes fs ablation of a CdSe target with UV pulses in pure water or methanol, gives some evidence of a weak yellow emission. 

We started by ablation with both 355 and 532 nm pulses, in methanol or pure deionized water. In all cases we could observe formation of pale yellow suspensions, which thoroughly precipitated within a few hours. The addition of PAMAM G5 to the initial solution permitted to obtain stable yellow-orange suspensions in the case of 532 nm ablation and aqueous environment. In contrast, either 355 nm pulses in PAMAM G5/water or 532 nm pulses in PAMAM G5/methanol did not allow to prepare stable samples. 

**Figure 10:**
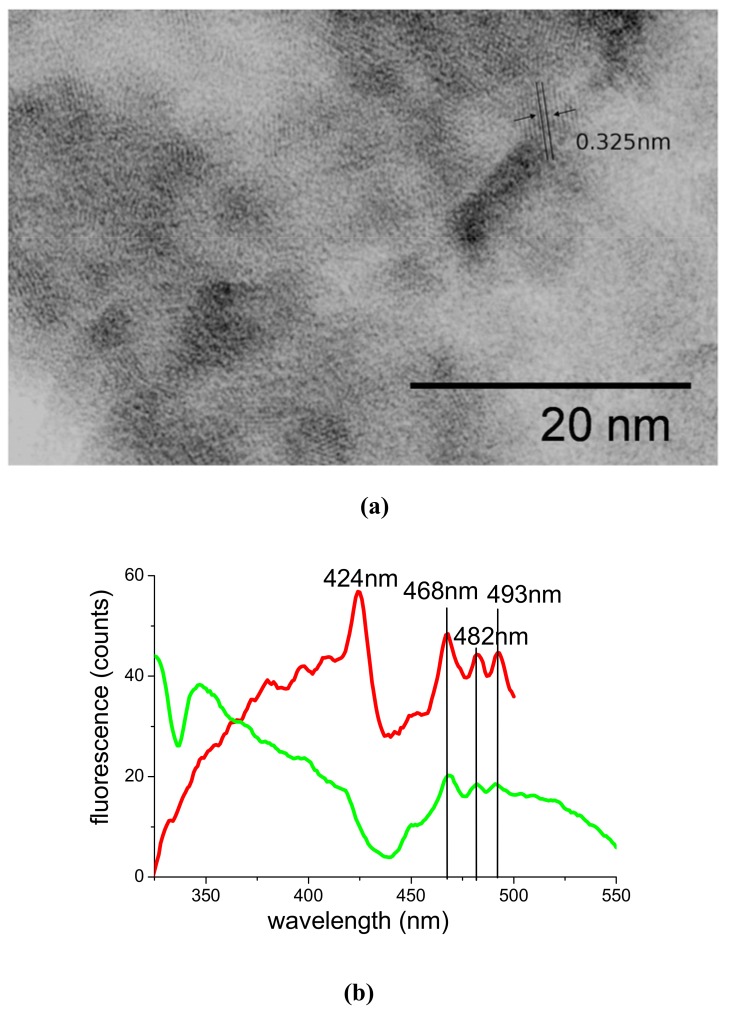
TEM image **(a)** and fluorescence spectra **(b)** of PAMAM G5-capped CdSe QDs. Excitation of the fluorescence is obtained with 270 nm (red curve) or 300 nm (green curve).

The results obtained by 532 nm ablation with 10 mJ pulses and three hours irradiation are reported in Figures 10a,b. To our knowledge, they represent the first report of either ps ablation of CdSe and use of PAMAM as its capping stabilizer. Figure 10a shows a TEM picture of one of our samples. The microanalysis performed during TEM microscopy of our samples gave an excess of Se, which is in agreement with the stechiometry of cadmoselite. Concerning particle dimensions, we can estimate an average size in the 3-4 nm range from TEM images. Crystalline planes are clearly visible and measurable and correspond to a period of 0.325 nm, which is consistent with the zincblende phase of the material. Indeed, CdSe can be found in the cubic zincblende phase [35] or in the hexagonal wurzite phase, with larger lattice period [37].

Fluorescence spectra of the samples are illustrated by Figure 10b. They were obtained by subtracting the fluorescence of a PAMAM G5/water solution exposed to the same irradiation process used for the ablation. The most efficient excitation line was 270 nm. However, an appreciable emission was also recorded under 300 nm excitation. Three characteristic peaks at 468, 482 and 493 nm can be observed with both exciting wavelengths, while the most intense one at 424 nm is observed by excitation with 270 nm only. The existence of multiple peaks must be attributed to the coexistence of different particle dimensions in the same sample, while their spectroscopic position in blue – deep blue spectral range is consistent with the small dimension of our particulate.

## 3. Experimental 

Nanoparticles were prepared by laser ablation of metallic or semiconductor targets in water. We used the fundamental (1,064 nm), second (532 nm) or third harmonic (355 nm) of a mode-locked Nd-YAG laser (EKSPLA PL2143A: rep. rate 10 Hz, pulse width 25 ps at 1064 nm, 20 ps at 532 nm and 15 ps at 355 nm). We post irradiated the obtained suspensions by using the same laser, namely the third harmonic or the second harmonic. The pulse energy was varied from 4 to 20 mJ and the ablation or post-irradiation time from few minutes to several hours. When not otherwise specified, the focussing conditions of the laser beam were maintained constant and the diameter of the laser spot on the target was fixed at 1.4 mm. The fluence on target was varied from 0.1 to 2 J/cm^2^. The target was placed in a 1cmx1cm quartz cuvette and was kept 2 cm in front of the focal plane of the laser beam. We used 2 mL of stabilizing solution for ablation and 1 or 2 mL for post-irradiation tests. 

The ablation process and the subsequent photofragmentation of nanoparticles were monitored by measuring on-line the visible spectra with an Ocean Optics fiber spectrophotometer and a tungsten lamp**.** The sampling beam was perpendicular to the ps laser beam and crossed the quartz cuvette 0.5 cm above the bottom of the cell.

We prepared AuNPs suspensions in solutions of PAMAM G5 in water. The solutions were 3.8 mM with regards to superficial amino groups and were obtained by dilution with ultrapure water (18.2 MΩ^.^cm @ 25 °C) of 22.2% or 6.37% aqueous solution of PAMAM G5 purchased from Dendritech^®^. According to tabulated values provided by the producer, PAMAM G5 is a monodisperse compound, having a molecular diameter of 5.4 nm. The gold and silver targets were purchased from Goodfellow and the crystalline CdSe target was purchased from Cradley Crystals. As declared by the producer, the crystal was not exactly stechiometric, but contained an excess of Cd, corresponding to 10^16^-10^17^ atoms/cm^3^.

We recorded UV-Vis spectra one day after the preparation of the suspensions with a double beam spectrophotometer (Perkin Elmer mod. Lambda19) and fluorescence spectra with a Jasco FP-750 spectrofluorimeter. The particles’ mean diameter and dispersivity were determined by TEM analysis. TEM samples were obtained by dipping carbon-coated copper grids in the suspensions and the images were recorded with a HRTEM JEOL2010, 200KV.

## 4. Conclusions

We have reviewed our experimental results concerning the use of dendrimer PAMAM G5 as a stabilizing agent in the production of Au, Ag and CdSe nanoparticles obtained by ps laser ablation of a solid target in liquid environment. Indeed, to our knowledge, this molecule, which is extensively employed to cap metal nanoparticles obtained by chemical reduction methods, has never been used in other laboratories for laser ablation tests. The chemical characteristics of PAMAM G5 permit efficient stabilization of the obtained samples. In particular, in the case of CdSe, we have shown for the first time that the presence of the dendrimer in the solution permits preparation of stable and fluorescent samples, corresponding to the zincblend phase of the material. 

Concerning preparation of PAMAM G5 capped AuNPs, we have discussed the role of ablating wavelength and pulse energy. We have shown that the use of the dendrimer permits stabilization of the different products formed by the interaction of the laser pulse with the target or with pre-formed nanoparticles, thus providing a deep insight into the ablation process. In particular, the presence of PAMAM G5 in AuNPs suspensions allows trapping and immobilization of sub-nanoclusters and cations and consequent monitoring of the fragmentation caused by irradiation of AnNPs with 532 and 355 nm pulses. While the presence of sub-nanoclusters can be demonstrated by proper fitting of the UV-Vis spectra of the suspensions, the presence of Au^3+^ is evidenced by the growth of a LMCT band in the UV region of the absorption spectrum. Moreover, the formation of Au^3+^, and its interaction with the dendrimer, causes an impressive increase of PAMAM G5 fluorescence. Although it is still unclear which part of the molecule acts as colour centre, we believe that the emission of our samples does not originate from gold, but it is to be thoroughly assigned to PAMAM through its Au^3+^–catalized oxidation. 

Lastly, the stabilizing properties of PAMAM G5 permit reshaping of AuNPs by post-irradiation methods, that is tailoring of their average size and dispersivity. This effect can be observed also in the case of Ag. Indeed, we have shown that AgNPs can also be efficiently stabilized by the dendrimer and that, under proper conditions, they can undergo a photofragmentation process, which permits preparation of stable suspensions of nano or sub-nano particles with reduced dimensions. 

## Figures and Tables

**Table 1:** Deconvolution of the fluorescence spectra of Figure 8.

Excitation wavelength (nm)	Emission wavelength (nm)	Emission wavelength (nm)	Emission wavelength (nm)	Emission wavelength (nm)	Emission wavelength (nm)
**PAMAM G5**					
400			424 (1%)	473 (5%)	480 (94%)
350		399 (11%)	416 (12%)	454 (13%)	469 (65%)
300	374 (26%)	399 (15%)	423 (9%)	466 (45%)	516 (5%)
**PAMAM G5 after 532nm irradiation**					
400			425 (1%)	470 (5%)	482 (94%)
350		401 (10%)	418 (6%)	457 (12%)	460 (71%)
300	359 (7%)	393 (38%)	426 (2%)	462 (35%)	515 (16%)
**PAMAM G5/AuNPs after 532nm bleaching**					
400		423 (3%)	470 (23%)	470 (70%)	516 (4%)
350		395 (11%)	416 (7%)	454 (78%)	463 (4%)
300	378 (20%)	398 (20%)	422 (6%)	468 (49%)	510 (4%)

## Appendix: Simulation of UV-Vis spectra

Due to the small size of our AuNPs, the dipole approximation of Mie’s cross section holds true and hence the extinction cross section, at a frequency ω of a AuNP whose radius is *R_NP_*, reads [21]:

(1) $$\sigma_{Mie}\left(\omega ,R_{NP}\right)=9\frac{\omega }{c}\varepsilon_{M}^{3/2}\frac{4\pi R_{NP}^{3}}{3}\frac{\varepsilon_{IM}\left(\omega ,R_{NP}\right)}{{\left[\varepsilon_{RE}\left(\omega ,R_{NP}\right)+2\varepsilon_{M}\right]}^{2}+\varepsilon_{IM}{\left(\omega ,R_{NP}\right)}^{2}}\;{\mathrm{cm}}^{2}$$

where the subscripts RE and IM refer to the real and imaginary part of the dielectric constant of the metal, which is corrected compared with the bulk value by size-dependent dumping terms, namely:

(2) $$\varepsilon_{RE}\left(\omega ,R_{NP}\right)=\varepsilon_{RE\infty }\left(\omega ,R_{NP}\right)+\omega_{p}^{2}\left(\frac{1}{\omega^{2}+\Gamma_{\infty }^{2}}-\frac{1}{\omega^{2}+\Gamma {\left(R_{NP}\right)}^{2}}\right)$$

(3) $$\varepsilon_{IM}\left(\omega ,R_{NP}\right)=\varepsilon_{IM\infty }\left(\omega ,R_{NP}\right)+\frac{\omega_{p}^{2}}{\omega }\left(\frac{\Gamma \left(R_{NP}\right)}{\omega^{2}+\Gamma {\left(R_{NP}\right)}^{2}}-\frac{\Gamma_{\infty }}{\omega^{2}+\Gamma_{\infty }^{2}}\right)$$

where the subscript ∞ refers to the bulk values. Γ(R_NP_) is the size-dependent relaxation frequency given by:

(4) $$\Gamma \left(R_{NP}\right)=\Gamma_{\infty }+\Psi \frac{V_{F}}{R_{NP}}$$

V_F_ is the Fermi velocity and Ψ is a coefficient of the order of 1 [21]. Finally, *ε_M_* is the real dielectric constant of the embedding medium. To calculate the absorption coefficient *A(ω)*, Equation 1 must be integrated over the normalized size-distribution *G(R)* of the NPs, i.e.:

(5) $$A\left(\omega \right)=D_{NP}^{}\int_{0}^{\infty }G\left(R\right)\sigma_{Mie}\left(\omega_{L},R\right)dR\;{\mathrm{cm}}^{-1}$$

where *D_NP_* is the density of NPs.

In principle, the best fit of the absorbance by Equation 5 with Mie’s cross section of Equation1 would lead to determine *D_NP_* by using the size-distribution obtained by TEM analysis. Nevertheless, we observed that this simple scheme required substantial refinement to account for the recorded spectra. Basically, discrepancies arose from the value of *ε_M_* and from the portion of spectrum at wavelengths shorter than the plasmon resonance.

Indeed, once the size-distribution is known, the wavelength of the plasmon resonance depends only on *ε_M_*. Our plasmon spectra were fitted well by assuming *ε_M_* = 2.2-2.6 while *ε_M_* had to be 1.77, being the particles dispersed in water. Therefore, we adopted the so-called core-shell approach to account for the absorption spectra, by assuming that the AuNPs are coated by a 5.4 nm-thick-PAMAM G5 shell. The core-shell quasi-static approximation for the polarizability *α(ω)* under dipolar approximation reads [21]:

(6) $$\alpha \left(\omega ,R_{NP}\right)=\frac{4\pi }{3}{\left(R_{NP}+d\right)}^{3}\left[\frac{\left(\varepsilon_{s}-\varepsilon_{M}\right)\left(\varepsilon +2\varepsilon_{s}\right)+{\left(\frac{R_{NP}}{R_{NP}+d}\right)}^{3}\left(\varepsilon -\varepsilon_{s}\right)\left(\varepsilon_{M}+2\varepsilon_{s}\right)}{\left(\varepsilon_{s}+2\varepsilon_{M}\right)\left(\varepsilon +2\varepsilon_{s}\right)+{\left(\frac{R_{NP}}{R_{NP}+d}\right)}^{3}\left(\varepsilon -\varepsilon_{s}\right)\left(2\varepsilon_{s}-2\varepsilon_{M}\right)}\right]$$

where d is the shell thickness, *R_NP_* is the core radius (radius of AuNP surrounded by the PAMAM shell), ε_s_ is the complex dielectric function of the shell, ε is the core (AuNP) complex dielectric function and ε_M_ the real dielectric function of the embedding medium. We have assumed ε_s_ to be real since the absorption of PAMAM G5 in the plasmon region is negligible. The extinction cross section is then given by:

(7) σMieCS(ω,RNP)=4πωcIm[α(ω,RNP)]

The absorption coefficient is simply obtained by inserting the core-shell extinction cross section in equation 5, in place of the value given by Equation 1. By this model we could solve the discrepancy of the embedding dielectric constant. In fact, plasmon resonance curves are now fitted well with ε_M_ = 1.77 (aqueous embedding medium), ε_s_ = Re(ε_s_) = 2.2-2.6 and d = 5.4 nm, i.e., the size of the PAMAM G5 shell. Therefore, the displacement towards the red of plasmon resonance, with respect to the spectral position expected for water solutions, is due to the dielectric constant of the dendrimer shell.

Although the core-shell model allowed us to solve the apparently anomalous value of the dielectric constant of the medium, it does not change the shape of the absorption curve obtained by using Equation 5, which remains systematically lower than the experimental one in the blue-UV spectral range (see Figure 5). To account for this extra-absorption, we introduced the contribution of particles whose size is small enough that the plasmon oscillations are strongly damped by quantum effects [21]. Their absorption looks like the bulk one, i.e. it increases almost monotonically towards the blue side of the spectrum (see Figure 5). To reproduce this feature, we assigned a value for the coefficient Ψ appearing in Equation 4, which is large enough to completely damp the plasmon resonance. Typically, we used Ψ = 10^2^, but any value above 10 is good and not critical. The contribution to the total absorption is then given by a term similar to Equation 5, but characterized by an extinction coefficient which contains a strong damping. In this respect, use of Equations 1 or 6 leads to the same results since the plasmon resonance disappears. Therefore, since Mie’s cross section turns out to be independent of R_NP_, the coefficient of Equation 5 corresponds to the total number density of sub-nm particles regardless of its distribution. For the sake of simplicity, we ascribe to atoms the number density value which gives the appropriate correction to fit the whole absorption spectrum.

Finally, we used the following procedure to fit the experimental curves: First, we fitted the plasmon resonance and the red-side part of spectrum by using Equations 5 and 6. As previously stressed, reliability of the fit requires a preliminary knowledge of the size-distribution from TEM analysis. Note that same results could be obtained by Equation 1 provided that an artificial value for ε_M_ > 1.77 is used. From the best fit, we first obtain the density D_NP_ of AuNPs. Figure 5 confirms that after such fitting there is a marked difference in the blue-side absorption albeit the plasmon curve and its red-side are accounted very well. Such discrepancy is then corrected by adding the contribution of atoms and clusters multiplied by an appropriate factor which in turn corresponds to D_A._
